# Autoantibodies Against Carbonic Anhydrase I and II in Patients with Acute Myeloid Leukemia

**DOI:** 10.4274/tjh.2016.0341

**Published:** 2017-12-01

**Authors:** Ahmet Menteşe, Nergiz Erkut, Selim Demir, Serap Özer Yaman, Ayşegül Sümer, Şeniz Doğramacı, Ahmet Alver, Mehmet Sönmez

**Affiliations:** 1 Karadeniz Technical University Vocational School of Health Sciences, Program of Medical Laboratory Techniques, Trabzon, Turkey; 2 Karadeniz Technical University Faculty of Medicine, Department of Hematology, Trabzon, Turkey; 3 Karadeniz Technical University Faculty of Health Sciences, Department of Nutrition and Dietetics, Trabzon, Turkey; 4 Karadeniz Technical University Faculty of Medicine, Department of Medical Biochemistry, Trabzon, Turkey; 5 Recep Tayyip Erdoğan University Faculty of Health Services, Department of Nursing, Rize, Turkey; 6 Recep Tayyip Erdoğan University Faculty of Medicine, Department of Medical Biochemistry, Rize, Turkey

**Keywords:** Acute myeloid leukemia, Autoantibody, Cancer, Carbonic anhydrase

## Abstract

**Objective:**

Cancer, one of the principal causes of death, is a global social health problem. Autoantibodies developed against the organism’s self-antigens are detected in the sera of subjects with cancer. In recent years carbonic anhydrase (CA) I and II autoantibodies have been shown in some autoimmune diseases and carcinomas, but the mechanisms underlying this immune response have not yet been explained. The aim of this study was to evaluate CA I and II autoantibodies in patients with acute myeloid leukemia (AML) and to provide a novel perspective regarding the autoimmune basis of the disease.

**Materials and Methods:**

Anti-CA I and II antibody levels were investigated using ELISA in serum samples from 30 patients with AML and 30 healthy peers.

**Results:**

Anti-CA I and II antibody titers in the AML group were significantly higher compared with the control group (p=0.0001 and 0.018, respectively). A strong positive correlation was also determined between titers of anti-CA I and II antibodies (r=0.613, p=0.0001).

**Conclusion:**

Our results suggest that these autoantibodies may be involved in the pathogenesis of AML. More extensive studies are now needed to reveal the entire mechanism.

## Abstract (tr)

**Amaç:**

Kanser, dünyadaki başlıca ölüm nedenlerinden birisi olup, küresel bir toplum sağlığı sorunudur. Organizmanın kendi antijenlerine karşı gelişen otoantikorlar pek çok kanser hastasının serumunda tespit edilmiştir. Son yıllarda karbonik anhidraz (KA) I ve II otoantikorlarının varlığı bazı otoimmün hastalıklarda ve kanser türlerinde gösterilmiştir, ancak bu immün yanıtın altında yatan mekanizmalar henüz açıklanabilmiş değildir. Bu çalışmanın amacı, akut miyeloid lösemili (AML) kişilerde, KA I ve II otoantikorlarının varlığını değerlendirmek ve hastalığın otoimmün temeline dair yeni bir bakış açısı sağlamaktır.

**Gereç ve Yöntemler:**

Otuz hasta ve 30 sağlıklı kontrolden elde edilen serum örneklerinde anti-KA I ve II antikor düzeyleri ELISA yöntemiyle belirlendi.

**Bulgular:**

AML grubundaki anti-KA I ve II antikor düzeyleri kontrol grubu (p= sırasıyla 0,0001 ve 0,018) ile karşılaştırıldığında anlamlı derecede yüksek bulundu. Ayrıca KA I ve II otoantikor seviyeleri arasında güçlü bir pozitif korelasyon saptandı (r=0,613; p=0,0001).

**Sonuç:**

Elde edilen sonuçlar bu otoantikorların AML patogenezinde rolü olabileceğini düşündürmektedir. Kesin mekanizmayı ortaya çıkarabilmek için daha kapsamlı çalışmalar gereklidir.

## INTRODUCTION

Cancer is the second most important cause of mortality and a major public health problem worldwide [1]. Acute myeloid leukemia (AML) is a complex and particularly heterogeneous clonal disease involving arrest of differentiation in the myeloid lineage along with deposition of immature progenitors in bone marrow, thus concluding in hematopoietic failure [2]. The pathogenesis of AML involves various disorders, such as mutations in transcription factors or epigenetic modifiers, aberrant signaling pathways, excessive expression of the gene involved in multidrug resistance, abnormal immune function, and abnormalities in the bone marrow microenvironment [3]. Malignant diseases progress with the stimulation of autoimmunity, characterized by the formation of antibodies against their own antigens. Autoantibodies can be observed in the sera of patients with solid tumors and hematological malignancies [4,5]. These autoantibodies are regarded as early biomarkers for some types of cancer [6,7,8].

Carbonic anhydrases (CAs) are vitally important enzymes responsible for the regulation of acid-base homeostasis in both healthy and pathological conditions. Members of the CA family contain 16 isoenzymes that differ from one another in terms of tissue distribution, cell localization, catalytic activity, and resistance to inhibitors. They perform several functions, such as transport of carbon dioxide, pH regulation, ion transport, formation of stomach acidity, bone resorption, calcification, and tumorigenesis during cancer cell development and invasion [9,10]. CA I and II are both cytosolic enzymes present in significant numbers in erythrocytes. CA I is the second most plentiful protein in erythrocytes after hemoglobin. CA II is a highly active isoenzyme involved in much total CA activity in a number of tissues. CA I and/or II autoantibodies have recently been demonstrated in various pathological conditions, such as autoimmune diseases (systemic lupus erythematosus, primary biliary cirrhosis, rheumatoid arthritis, and Sjögren’s syndrome) and carcinomas (lung, colon, and prostate). However, the mechanisms underlying this immune response have not yet been explained [11,12,13,14]. The purpose of this study was to investigate CA I and II autoantibodies in patients with AML and to provide a novel perspective regarding the autoimmune basis of the disease.

## MATERIALS AND METHODS

### Study Group

Informed consent was obtained from all patients and controls. Approval for the study was granted by the local ethics committee. Thirty patients newly diagnosed with AML were included as the study group and 30 healthy peers as the control group. Diagnosis of AML was made and verified by a panel of hematologists who also classified each case according to the French-American-British (FAB) classification [15]. The subtypes of AML according to FAB were as follows: M0: 1 (3.3%); M1: 1 (3.3%); M2: 13 (43.3%); M3: 3 (10%); M4: 9 (30%); M5: 2 (6.6%); M6: 1 (3.3%). Patients were selected from individuals presenting to the hematology clinic and referred from other practitioners. The study group consisted of 17 women and 13 men with a mean age of 52.8±6.3 years, and the control group of 17 women and 13 men with a mean age of 51.9±14.1. Patients with renal, coronary, or liver failure and chronic inflammatory diseases or anemia, and subjects receiving chemotherapy or using oral contraceptives and anticoagulants, were excluded from the study.

Blood samples of 5 mL from each individual were placed into vacutainer tubes without anticoagulant. These were then centrifuged at 1800xg for 10 min. Serum samples were stored at -80 °C until being used for measurements. Platelet (PLT), hemoglobin (Hb), hematocrit (Hct), and white blood cell (WBC) levels were determined with a Beckman Coulter autoanalyzer.

### Determination of Serum Autoantibody to CA I and II

ELISA is frequently used to detect autoantibodies in blood samples since it is economical, simple, and quick to perform [16,17]. It has also been widely used for the evaluation of CA I and II autoantibodies in different pathological conditions in previous reports [12,13,18,19,20,21]. Serum CA I and II autoantibodies were therefore determined using the ELISA method as previously described elsewhere [18]. Briefly, flat-bottomed plates were coated with CA I or II (10 µg/mL) (Sigma-Aldrich, St. Louis, MO, USA) in carbonate buffer (pH 9.6). These were then incubated for 18 h at 4 °C. In the next stage, the wells were washed four times with phosphate buffer (PBS) (pH 7) before being blocked with 3% skim milk in PBS at room temperature for 2 h. The wells were next washed again four times with PBS containing 0.05% Tween-20 before incubation with 100 µL of 1:200 diluted serum for 2 h. Following these washing procedures, each individual well was incubated for 2 h with 100 µL of a 1:2000 solution of peroxidase-conjugated anti-human IgG anti-serum (Sigma-Aldrich, St. Louis, MO, USA) in 3% skim milk in PBS. A further five washes were performed with PBS containing 0.05% Tween-20, and the wells were then incubated with 100 µL of substrate solution for 20 min. Reactions were halted by adding 100 µL of 2 M sulfuric acid to each well. The resulting absorbance was measured at 480 nm (Molecular Devices, Sunnyvale, CA, USA). Control wells containing no CA I or II were also employed for ELISA investigation of each serum studied. All assays were performed in duplicate. The specific binding of serum antibody to CA II was calculated as the mean absorbance of the antigen-coated wells minus the mean absorbance of the control wells. The results were expressed as absorbance units.

### Statistical Analysis

Data are shown as mean ± standard deviation for normally distributed and median (interquartile range) for non-normally distributed variables. Statistical analysis was performed with SPSS 13.0 (Chicago, IL, USA) and MedCalc (Version 12.3, Mariakerke, Belgium) statistical software. Compatibility with normal distribution was determined using the Kolmogorov-Smirnov test. Differences between the two groups were analyzed using Student’s t-test for normally distributed data. Correlation analysis was calculated using Pearson’s correlation coefficient and the nonparametric equivalent Spearman’s rank correlation coefficient at a 95% confidence interval. Receiver operating characteristic (ROC) curves were used to detect the discriminatory dominance of CA I and II autoantibodies for the identification of AML. Sensitivity, specificity, negative predictive value (NPV), and positive predictive value (PPV) were determined from ROC graphs for autoantibodies of CA I and II. p<0.05 was regarded as significant.

## RESULTS

**Figure 1:**
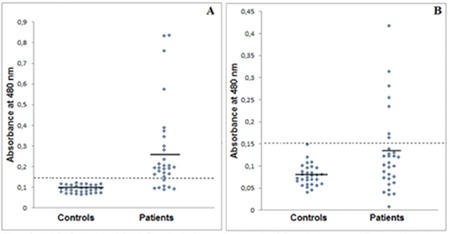
Anti-CA I (A) and anti-CA II (B) antibodies in sera of patients with acute myeloid leukemia and healthy controls. The dotted line indicates plus 3 SD of health control sera (A_480_=0.146 ABSU for anti-CA I antibody, A_480_=0.151 for anti-CA II antibody). ABSU: Absorbance units, SD: standard deviation, CA: carbonic anhydrase.

Thirty AML patients and 30 healthy subjects were included in this study. There was no significant difference in terms of mean age between the study and control groups. Levels of anti-CA I and II antibodies in patients with AML and control subjects are shown in Figure 1A and Figure 1B, respectively.

The mean absorbance value of the AML group was significantly higher (p=0.0001) than that of the healthy subjects (Table 1). The mean absorbance +3 standard deviations (SD) of healthy subjects was determined as positive. The mean absorbance value of anti-CA I antibody for healthy subjects was 0.092±0.018, and the absorbance was higher than 0.146. Positive results were obtained in 23 of the 30 cases of AML (Figure 1A). The mean absorbance value of the AML group was significantly higher (p=0.018) than that of the healthy controls (Table 1). The mean absorbance +3SD of healthy subjects was also positive. The mean absorbance value of anti-CA II antibody for the healthy subjects was 0.079±0.024, and the absorbance was higher than 0.151. Positive results were obtained in 7 of the 30 cases of AML (Figure 1B). We also observed a strong positive correlation between titers of anti-CA I and II antibodies (r=0.613, p=0.0001).

**Figure 2:**
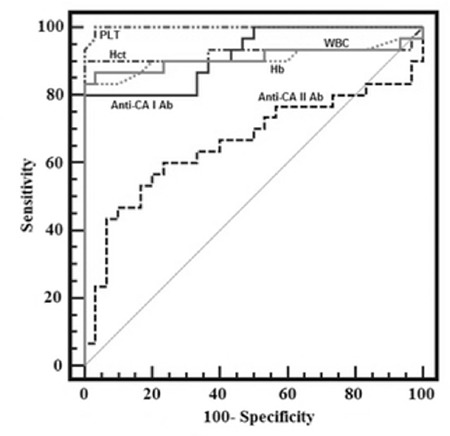
ROC curve analysis for all parameters in patients with acute myeloid leukemia. CA: Carbonic anhydrase, Hct: hematocrit, PLT: platelet, WBC: white blood cell.

ROC curve analysis was also used to quantify serum Hb, Hct, PLT, WBC, and anti-CA I and II levels. Values for cut-off points, area under the curve, sensitivity, specificity, PPV, and NPV for individual parameters are shown in Table 2 and Figure 2.

## DISCUSSION

Cancer is the second most important cause of mortality, and millions of people either have or have had the disease. An estimated 1.68 million new cancer cases and 595690 deaths from cancer are predicted to have occurred in the United States in 2016. Leukemia is one of the most common forms of cancer [1]. AML is a heterogeneous disease with marked malignancy of hematopoietic progenitor cells committed to the myeloid lineage. This phenomenon is most common in subjects aged over 70, and AML constitutes approximately 30% of all cases of leukemia [1,22]. Several mutated or overexpressed proteins seem to be processed and presented to the immune system as tumor antigens, leading to humoral and/or cellular responses [23]. Autoimmunity is well known to be potentially associated with cancer, and one of the forms of its expression is the development of autoantibodies and eventually autoimmune disease. Detection of autoantibodies may therefore be the first sign of cancer [24]. The ideal tumor biomarker would make it possible to detect cancer with a simple blood test. The serum biomarkers available today are based on the measurement of cancer antigens, such as prostate-specific antigen, carcinoembryonic antigen, the cancer antigens (CA15-3, CA19-9, and CA125), extracellular protein kinase A, anti-oncoprotein (HER-2/neu), anti-tumor suppression antigen (p53), anti-proliferation associated antigens (cyclin A, cyclin B1, and CDKs), anti-onconeural antigens (Hu and Yo), and anti-cancer/testis antigens (NY-ESO-1 and MAGE-1) [4,25]. There has therefore been considerable research in recent years into the identification of new biochemical diagnostic markers for the early detection of AMLs [3,22,26,27]. Analysis of serum autoantibodies may become a useful tool for clinicians in screening for cancer and diagnosis of AML. However, these markers exhibit limited specificity and sensitivity, and their levels can also rise even under benign conditions or during gestation. There is therefore an urgent need for novel biomarkers capable of adoption into routine clinical use in the diagnosis of AML and other cancer types [25].

Autoantibodies are common in cancer patients. The autoantibody response in AML patients has been considered in previous studies, such as Wilms tumor gene product [28], single-stranded DNA [29], anti-cardiolipin antibodies [30], the M-phase phosphoprotein 11 (MPP11) [31], receptor for hyaluronan acid-mediated motility (RHAMM) [32], and RHAMM-like protein [33]. This study is the first report to show an increased autoimmune response to both CA I and II in the sera of AML patients. The prevalence of CA I and II autoantibodies in patients with AML in this study was 76.6% and 23.3%, respectively. The presence of autoantibodies against CA I and II has been observed in many pathological conditions, such as metabolic syndrome, recurrent pregnancy loss, acute anterior uveitis, gastric cancer, Graves’ disease, preeclampsia, and rheumatoid arthritis. The prevalence of CA I autoantibody is reported in the range of 9.6%-20%, and that of autoantibodies against CA II in the range of 4.6%-72.5% [13,19,20,34,35]. From this perspective, our results were consistent with the literature.

Protection of the acid-base balance is of considerable importance in tumorigenesis. Extracellular hydrogen ion concentrations in solid tumors are reported to be higher than those in normal neighboring tissues [11,36]. Tumor cells express ion transport protein, such as vacuolar-type H+-ATPase, Cl-/HCO3-, and Na+/H+ exchangers between the inner and outer regions of the cell, thus creating a pH gradient. Many tumor cells synthesize CAs that catalyze the production of H+ and HCO3- ions [11]. CAs are currently the subject of significant research into carcinogenesis and tumor invasion. Studies have recently shown an incremental expression of specific cytosolic CA I and II in some carcinomas, including leukemia, and in the blast cells of AML [11,36,37]. CA I and II interact with various molecules due to their cellular localizations, functions, and wide tissue distribution. These proteins are therefore becoming target molecules in the body. Autoantibodies against CA I and II have recently been demonstrated in many pathological conditions, such as cancer, and autoimmune and idiopathic diseases. Although the mechanisms involved have not been identified exactly, oxidative stress has been reported to be potentially significant in the formation of these autoantibodies [13,14,19,21,38,39]. Oxidative stress results from acceleration of the rate of free radical formation and/or a decrease in the rate at which these are eliminated. In either condition a severe imbalance occurs between free radical formation and the antioxidant defense mechanism [40]. Increased reactive oxygen or nitrogen species (ROS) lead to tissue injury and compromise numerous biomolecules, including proteins, nucleic acids, structural carbohydrates, and lipids. The reaction of ROS with lipids causes these molecules to undergo oxidative breakdown. Malondialdehyde (MDA) is a one-end product of lipid peroxidation capable of being covalently bound to proteins, and especially to the Ɛ-amino groups of lysine residues. These oxidative disturbances may influence the immune system, resulting in the development of specific autoimmune processes [41]. The lipid peroxidation end-products 4-hydroxy-2-nonenal (HNE) and MDA are known to alter proteins and to modify their antigenic properties [42]. One study of erythrocytes proved that CA II is the first target of HNE [43]. Numerous anti-MDA-modified proteins have been detected in systemic diseases, such as systemic lupus erythematosus, periarteritis nodosa, scleroderma, atherosclerosis, and rheumatoid arthritis in previous studies. It has also been suggested that these autoantibodies may be of predictive value for systemic diseases [41,44,45,46,47]. Studies in the literature have shown that the levels of such oxidative stress parameters as MDA, advanced oxidation protein products, 8-hydroxydeoxyguanosine, and protein carbonyl increase in the sera of patients with AML, while the activities of antioxidant enzymes such as superoxide dismutase, glutathione peroxidase, and monoamine oxidase decrease [48]. In light of these data, we anticipate that oxidative byproducts, including MDA, might generate the spread of neoantigens and confirm a potential association between autoimmunity and oxidative stress.

### Study Limitations

The major limitation of this study is the relatively small sample size of the patient and control groups.

## CONCLUSION

In conclusion, CA I and II autoantibody titers were significantly higher in subjects with AML compared to the controls. More extensive studies are now needed to reveal the entire mechanism involved.

## Figures and Tables

**Table 1:**
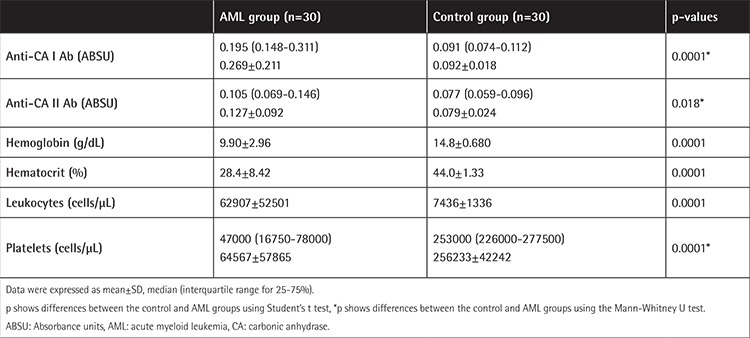
Clinical characteristics of the two groups.

**Table 2:**
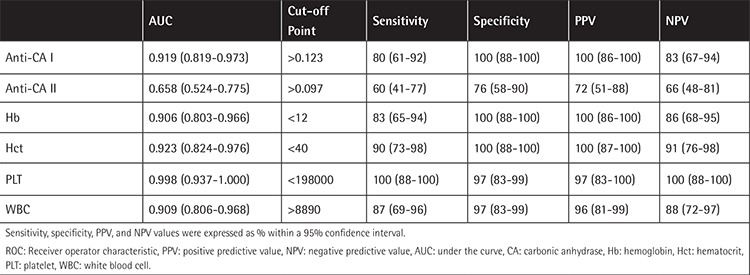
Receiver operator characteristic curve analysis of hemoglobin, hematocrit, platelet, white blood cell, and anti-carbonic anhydrase I and II antibody levels and their sensitivity, specificity, positive predictive value, and negative predictive value.
